# Changes to Blood-Sampling Protocol to Reduce the Sampling Amount in Neonatal Intensive Care Units: A Quality Improvement Project

**DOI:** 10.3390/jcm12175712

**Published:** 2023-09-01

**Authors:** Nayoung Jung, Chan Kim, Hanna Kim, Yekyeng Seo, Jieun Hwang, Misun Yang, So Yoon Ahn, Se In Sung, Yun Sil Chang

**Affiliations:** Department of Pediatrics, Samsung Medical Center, Sungkyunkwan University School of Medicine, Seoul 06351, Republic of Korea; 119youngna@gmail.com (N.J.); chan8788@naver.com (C.K.); khnhn111@gmail.com (H.K.); connie723@naver.com (Y.S.); abc907@naver.com (J.H.); hooray04@naver.com (M.Y.); yoon.ahn.neo@gmail.com (S.Y.A.); yunsil.chang@gmail.com (Y.S.C.)

**Keywords:** extremely low-birth-weight infants, blood sampling, blood transfusion

## Abstract

(1) Background: This study aimed to evaluate whether the implementation of a modified blood-sampling protocol, which focused on need-based laboratory testing and minimized venous sampling by replacing it with point-of-care testing (POCT) via capillary puncture, successfully reduced iatrogenic blood loss, incidence of anemia, and the frequency of blood transfusion among extremely low-birth-weight infants (ELBWIs) without negatively affecting neonatal outcomes. (2) Methods: A retrospective analysis was conducted on 313 ELBWIs with a gestational age (GA) of between 23 and 28 weeks and born between 2013 and 2019. The infants were divided into two groups corresponding to the periods before (period I) and after (period II) the implementation of the modified blood-sampling protocol in January 2016. Propensity score matching was conducted to minimize selection bias. Clinical data, including the frequency and amount of blood sampling, the frequency and volume of blood transfusion, and clinical characteristics, such as gestational age, birth weight, and neonatal outcome data, were collected and compared between the two groups. (3) Results: No significant differences in GA or birth weight between the two periods were observed. The total sampling volume a month after birth (16.7 ± 4.1 mL vs. 15.6 ± 4.4 mL, *p* = 0.03) and the total sampling volume during hospitalization days (51.4 ± 29.7 mL vs. 44.3 ± 27.5 mL, *p* = 0.04) in period II were significantly lower than those in period I. There were no differences in terms of anemia (hemoglobin 10.8 ± 2.2 vs. 11.0 ± 1.9, *p* = 0.43) and mortality or morbidity, such as intraventricular hemorrhage, retinopathy of prematurity, bronchopulmonary dysplasia, necrotizing enterocolitis, and sepsis, between the two periods. Although the transfusion frequency and amount did not present significant differences between the periods, we observed a positive correlation between the transfusion frequency and sampling volume (coefficient: 0.09, 95% CI: 0.08–0.11). (4) Conclusions: The modified blood-sampling protocol effectively reduced the level of iatrogenic blood loss without negatively affecting the neonatal outcomes.

## 1. Introduction

Premature infants have been observed to have a higher risk of developing anemia due to iatrogenic blood loss and physiological responses, often necessitating multiple blood transfusions during hospitalization [1]. Extremely low-birth-weight infants (ELBWIs) are particularly susceptible to iatrogenic anemia due to the relatively larger sampling volume required from them for frequent laboratory tests compared to their small total blood volume, shorter lifespan of blood cells, and immature erythropoietic response [2,3]. It is estimated that approximately 90% of ELBWIs will require at least one red blood cell transfusion; however, transfusions can lead to complications, mortality, and major neonatal morbidities, such as necrotizing enterocolitis and intraventricular hemorrhage, among premature infants. Therefore, a reduction in the blood-sampling frequency and volume is crucial for quality improvement in neonatal intensive care units (NICUs) [4,5].

In 2016, we implemented a change in our blood-sampling protocol from routinely scheduled sampling to a need-based schedule with a lower sampling volume. The present study aimed to determine whether this protocol change successfully reduced iatrogenic blood loss, incidence of anemia, and the frequency of blood transfusions for ELBWIs without negatively affecting their neonatal outcomes.

## 2. Materials and Methods

**Figure 1:**
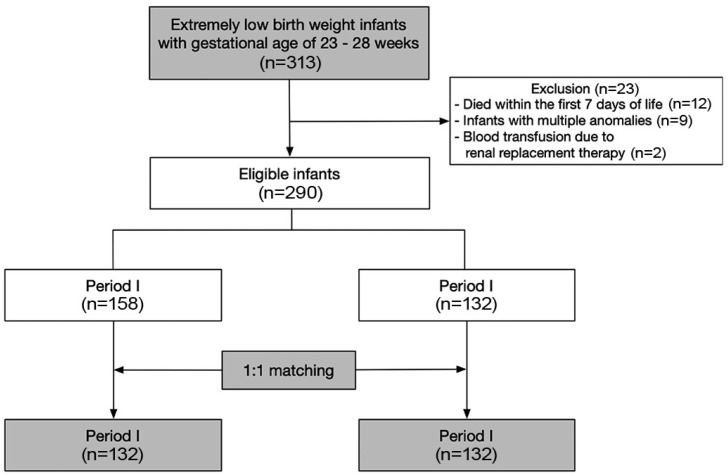
Enrollment flow diagram.

As part of a QI project, we implemented a modified blood-sampling protocol in January 2016. To assess the impact of this change, we retrospectively reviewed the medical records of 313 ELBWIs with a gestational age of between 23 and 28 weeks who were born and admitted to the Samsung Medical Center NICU between 2013 and 2019. The infants were divided into two groups based on their date of birth, i.e., before (period I) and after (period II) January 2016. Infants who died within the first seven days of life (*n* = 12), those with multiple congenital anomalies (*n* = 9), and those who received blood transfusions due to continuous renal replacement therapy (*n* = 2) were excluded from the analysis. Of the remaining 290 infants, 264 (132 per period group) were selected using propensity score matching to minimize selection bias (Figure 1).

During period I, the blood-sampling protocol involved routine scheduling, which encompassed (1) venous sampling for complete blood cell count (CBC), electrolytes, c-reactive protein (CRP), and chemistry profiles on postnatal days 1, 3, and 7 during the first week and, subsequently, once per week for infants with central parenteral nutrition and once every two weeks for infants without central parenteral nutrition, with a total blood volume of 1.2 mL per sampling for CBC, electrolytes, CRP, and chemistry profiles altogether, and (2) heel puncture for capillary blood gas analysis (CBGA) on a daily basis for infants under mechanical ventilation, with 0.1 mL per sampling. In period II, the protocol was modified to focus on needs-based laboratory testing and minimize venous sampling by replacing it with point-of-care testing (POCT) via capillary puncture. The modified protocol included (1) umbilical cord blood testing instead of venous sampling on day 1, excluding blood culture; (2) constructing a POCT-chemistry profile for blood urea nitrogen, creatinine, sodium, potassium, chloride, ionized calcium, lactate, total bilirubin, and micro-CRP, with a total volume of 0.1 mL per sampling, which was performed once every one or two weeks; (3) venous sampling for CBC and other chemistry profiles not included in POCT-chemistry, such as liver enzymes, triglycerides, and serum magnesium, which was performed based on the clinician’s decision; and (4) CBGA, which was performed only when clinically necessary. In a typical case involving mechanical ventilation, the cumulative volume of blood samples collected on postnatal day 7 was approximately 4.3 mL during period I, ranging from 1.5 to 2.7 mL in period II.

We conducted a monthly education program on the changed blood-sampling protocols for NICU physicians. Additionally, during rounds, we discussed the necessity of all blood sampling procedures either before or after they were performed. We transfused a blood volume of 10–15 mL/kg in a single transfusion and administered iron at a dosage of 2–4 mg/kg/day starting at four weeks of age. We maintained this regimen until discharge. This protocol remained unchanged throughout the study period.

After obtaining approval from the IRB, we manually reviewed medical records to extract data. We extracted various forms of clinical data from medical records, including the frequency and amount of blood sampling, frequency and volume of red blood cell (RBC) transfusions, and clinical characteristics, such as gestational age, birth weight, Apgar scores, and perinatal maternal history. The neonatal outcomes data included the incidence of intraventricular hemorrhage grades 3 and 4 based on Papile’s classification, retinopathy of prematurity (ROP) stage III or higher, necrotizing enterocolitis (NEC) stage 2b or higher based on modified Bell’s criteria, moderate-to-severe bronchopulmonary dysplasia (BPD) defined as oxygen dependency at a corrected age of 36 weeks, and culture-proven sepsis. The demographic characteristics, sampling frequency and volume, hemoglobin (Hb) and hematocrit (Hct) levels on postnatal days 30 and 60, frequency of RBC transfusions, and major neonatal morbidities were compared between the two groups (periods I and II).

Among the eligible infants (*n* = 290, Figure 1), we selected 132 infants per period using a propensity score analysis with 1:1 matching using the nearest matching method to minimize the bias stemming from differences in the clinical characteristics of the infants in the different study periods. The variables included in the propensity score analysis were gestational age, sex, antenatal steroid use, incidence of intraventricular hemorrhage grade 3–4, and moderate-to-severe bronchopulmonary dysplasia. After propensity matching, we compared periods I and II using the chi-square test for categorical variables and the *t*-test for continuous variables. Linear regression was conducted to evaluate the correlation between the cumulative blood sampling volume and transfusion frequency during hospitalization. We conducted data analysis using R version 4.2 (R Project for Statistical Computing).

## 3. Results

**Figure 2:**
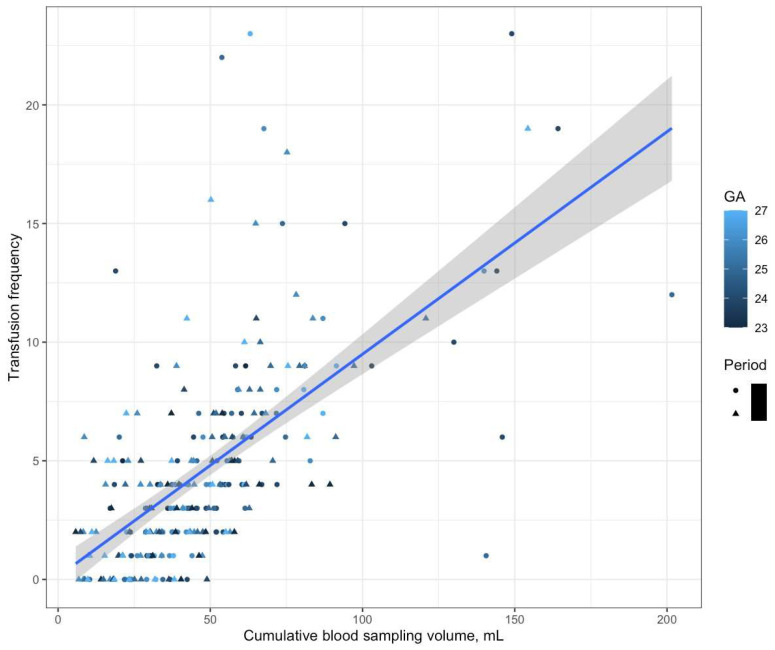
Positive correlation between blood sampling volume and the frequency of blood transfusion during hospitalization. GA: gestational.

Table 1 presents the demographic and clinical characteristics of the patients during each study period. There were no significant differences in antenatal corticosteroid use, maternal hypertension, or gestational age between the two periods. However, Apgar scores at 1 and 5 min were significantly higher in period II compared to period I (4.9 ± 1.5 vs. 4.3 ± 1.4 at 1 min, *p* < 0.001; 7.7 ± 1.2 versus6.8 ± 1.4 at 5 min, *p* < 0.001). The cumulative sampling volume until postnatal day 30 and the total cumulative sampling volume during hospitalization were both significantly lower in period II compared to period I (15.6 ± 4.4 mL vs. 16.7 ± 4.1 mL, *p* = 0.03; 44.3 ± 27.5 mL vs. 51.4 ± 29.7 mL, *p* = 0.04, respectively). Although Hb and Hct levels on day 30 were slightly higher in period II (10.8 g/dL vs. 11.0 g/dL; 31.7% vs. 32.7%), the difference was not statistically significant (Table 2). There was no difference in the frequency or cumulative volume of RBC transfusions during hospitalization between the two periods. However, the linear regression model showed a statistically significant positive correlation between the cumulative blood-sampling volume and transfusion frequency during hospitalization (Figure 2), with every 11 mL of blood sampled leading to one blood transfusion (coefficient: 0.09, 95% CI: 0.08–0.11). Regarding neonatal outcome variables, there were no differences between the two periods with respect to mortality (18% vs. 22%, *p* = 0.54), intraventricular hemorrhage (11.4% vs. 12.9%, *p* = 0.85), retinopathy of prematurity (35.6% vs. 39.4%, *p* = 0.61), bronchopulmonary dysplasia (47.0% vs. 59.1%, *p* = 0.06), necrotizing enterocolitis (12.9% vs. 17.4%, *p* = 0.39), and sepsis (23.5% vs. 27.3%, *p* = 0.57) (Table 3).

## 4. Discussion

In the present study, we were able to successfully reduce the amount of iatrogenic blood loss in ELBWIs by implementing a new QI project. Although the reduction in blood loss did not result in an increase in hemoglobin levels or a decrease in the frequency of transfusions, this might have been due to the low statistical power resulting from the small sample size, considering the evidence of a positive correlation between the cumulative blood loss volume and the frequency of transfusions (Figure 2). Additionally, because there were no differences in the neonatal outcome variables between the two periods, it can be inferred that the protocol change toward fewer laboratory tests did not have any adverse effects on patient safety.

Blood sampling for diagnostic purposes is the most common cause of anemia, and it is a significant contributor to the need for RBC transfusion among preterm infants [2,4,6]. According to a study by Blanchette and Zippursky, laboratory blood loss levels in the first six weeks of life were between 11 to 22 mL/kg per week; this is the equivalent of 15–30% of the circulating blood volume of very-low-birth-weight infants (<1500 g) [6]. Another study by Aboalqez et al. reported a median absolute amount of blood sampled 6.5 mL (IQR 12.3–21.1 mL) during the initial four weeks for very-low-birth-weight infants (<1500 g) [7]. Compared to previous studies, our data showed a similar amount of blood loss of 15.6 mL during the first four weeks in even smaller infants (<1000 g), which was achieved by the adoption of a needs-based sampling protocol with the active use of POCT.

Physiological anemia is a common phenomenon in term infants and is typically characterized by a drop in hemoglobin levels to 11.0 g/dL at the age of 2 months. However, preterm infants experience earlier and more substantial hemoglobin drops, resulting in a decrease from 9.5 mL/dL to 9.0 mL/dL [8]. Anemia of prematurity is caused by premature birth which occurs before placental iron transport and fetal erythropoiesis completion; low plasma levels of erythropoietin due to reduced production and accelerated catabolism; and iatrogenic blood loss caused by laboratory testing [9]. Thus, preterm infants require more RBC transfusions than mature infants [10]. The study by Jansen et al. showed that infants born at 24–28, 28–30, and 30–32 weeks of gestation had RBC transfusion rates of 94%, 62%, and 35%, respectively [11].

On the other hand, the relationship between RBC transfusion and mortality among ELBWIs is well established [3,12,13,14]. The underlying mechanism of this association is believed to be related to transfusion-related immunomodulation with a massive release of proinflammatory cytokines [15]. Furthermore, transfusions in ELBWIs are also known to be associated with an increased risk of several morbidities, including NEC, ROP, IVH, and BPD [16,17,18]. In a study by Su et al., authors could reduce the incidence of NEC by means of reducing the cumulative blood sampling volume for very-low-birth-weight infants [19]. Taken together, considering the adverse effects of transfusion, it is crucial to reduce iatrogenic blood loss for preterm infants. Our data indicate that the new QI project aiming to reduce iatrogenic blood loss is effective and feasible even for extremely premature infants. This result was achieved by carefully evaluating the necessity and timing of each laboratory blood draw and by actively using POCT.

The main limitation of this study consists of the negative results observed in the primary endpoint, which was a reduction in transfusion. Although we did observe a positive correlation between the sampling volume and transfusion frequency, we could not identify a significant difference in transfusion frequency in the period we analysis. Despite maintaining consistent transfusion indications throughout the duration of the study, we could not exclude the possibility of a more liberal approach to transfusions during period II. Also, because this study analyzed two different periods, there was a chance of bias regarding the overall improvement in perinatal care in period II compared with period I. Although there were no differences in gestational age, birth weight, and the percentage of small-for-gestational age infants between the two periods, the Apgar scores at 1 and 5 min were significantly higher in period II compared with period I, suggesting a temporal improvement in delivery management. This limitation stems from the retrospective nature of this study, highlighting the need for prospective randomized clinical trials in the future. Another reason for the lack of a difference in the blood sampling frequency or hemoglobin levels could be that the reduction in blood sampling volume might have been too small to present an effect [20]. While expressing blood amount in terms of volume/kg instead of as an absolute value would have been more appropriate, it have been unable to achieve and this stands as one of the limitations of this study. Despite these limitations, we speculate that if a greater reduction in blood-sampling amount is implemented, differences could be observed. Also, the value of this study remains significant due to the scarcity of studies focused on large populations of ELBW infants.

## 5. Conclusions

In conclusion, despite the limitations of this study, including the relatively small study population from a single center and the retrospective study design, our results show the effectiveness and feasibility of implementing the new QI project with respect to reducing iatrogenic blood loss without compromising patient safety, possibly resulting from fewer diagnostic intervention strategies for ELBWIs. Our data also suggest that careful evaluation of the necessity and timing of laboratory tests is vital to reduce the risk of transfusion for these infants.

## Figures and Tables

**Table 1:** Comparison of the demographic and clinical data between periods I and II.

	Period I (*n* = 132)	Period II (*n* = 132)	*p*-Value
**Maternal characteristics**			
Oligohydramnios, *n* (%)	27 (20.5)	33 (25.0)	0.46
Premature rupture of membrane, *n* (%)	53 (40.2)	52 (39.4)	1.00
Maternal hypertension, *n* (%)	22 (16.9)	29 (22.0)	0.40
Gestational diabetes, *n* (%)	8 (6.1)	3 (2.3)	0.22
Antenatal steroid, *n* (%)	126 (95.5)	126 (95.5)	1.00
**Neonatal characteristics**			
Gestational age	25.0 ± 1.27	26.1 ± 1.37	0.61
Birth weight (g)	719.4 ± 150.4	710.3 ± 156.6	0.80
Male, *n* (%)	67 (50.8)	66 (50.0)	1.00
Small for gestational age, *n* (%)	27 (20.5)	36 (27.3)	0.25
Hospital days, day	119.2 ± 56.3	125.1 ± 79.2	0.49
Apgar scores			
1 min	4.3 ± 1.4	4.9 ± 1.5	<0.001
5 min	6.8 ± 1.4	7.7 ± 1.2	<0.001

**Table 2:** Data related to anemia and blood transfusion for ELBWIs.

	Period I (*n* = 132)	Period II (*n* = 132)	*p*-Value
Hb/Hct *			
Hb on day 30 (g/dL)	10.8 ± 2.2	11.0 ± 1.9	0.43
Hct on day 30 (%)	31.7 ± 5.7	32.7 ± 5.9	0.21
Hb on day 60 (g/dL)	10.1 ± 2.2	10.1 ± 1.7	0.97
Hct on day 60 (%)	30.5 ± 5.8	30.9 ± 4.5	0.61
**Transfusion**			
Transfusion frequency, *n*	4.5 ± 4.4	4.3 ± 3.8	0.67
Transfusion volume, mL	81.7 ± 94.5	81.7 ± 108.9	0.99
**Blood sampling**			
Frequency within 30 days, *n*	28.7 ± 4.3	28.0 ± 5.1	0.20
Volume within 30 days, mL	16.7 ± 4.1	15.6 ± 4.4	0.03
Total frequency, *n*	76.1 ± 48.7	71.7 ± 45.1	0.45
Total volume, mL	51.4 ± 29.7	44.3 ± 27.5	0.04

* Hb: hemoglobin; Hct: hematocrit.

**Table 3:** Comparison of mortality and morbidities between period I and II.

	Period I (*n* = 132)	Period II (*n* = 132)	*p*-Value
Mortality, *n* (%)	24 (18.2)	29 (22.0)	0.55
IVH *, *n* (%)	15 (11.4)	17 (12.9)	0.85
ROP *, *n* (%)	47 (35.6)	52 (39.4)	0.61
BPD *, *n* (%)	62 (47.0)	78 (59.1)	0.06
NEC *, *n* (%)	17 (12.9)	23 (17.4)	0.39
Sepsis, *n* (%)	31 (23.5)	36 (27.3)	0.57

* IVH: intraventricular hemorrhage grades III-IV; ROP: retinopathy of prematurity ≥ III; BPD: bronchopulmonary dysplasia, moderate to severe; NEC: necrotizing enterocolitis stage ≥ 2b.

## Data Availability

Not applicable.

## Abbreviations

ELBWIs	extremely low-birth-weight infants
GA	gestational age
IVH	intraventricular hemorrhage
ROP	retinopathy of prematurity
NEC	necrotizing enterocolitis
BPD	bronchopulmonary dysplasia
NICUs	neonatal intensive care units
CBC	complete blood cell count
CRP	c-reactive protein
CBGA	capillary blood gas analysis
POCT	point-of-care testing
RBC	red blood cell
Hb	hemoglobin
Hct	Hematocrit
QI	Quality improvement
